# The Failure to Recognize Continuing Harm: Post-Separation Domestic Abuse in Child Contact Cases

**DOI:** 10.1177/10778012241243049

**Published:** 2024-04-01

**Authors:** Kieran Walsh

**Affiliations:** 1 Portsmouth Law School, 6697 University of Portsmouth, UK

**Keywords:** domestic violence, contact, harm, post-separation, courts, England and Wales

## Abstract

This article presents findings from a case file review of post-separation parenting cases in England and Wales. It first outlines that jurisdiction's legal framework relevant to these cases, before providing an overview of the findings relating to the profile of the cases and their outcomes. It then describes the types of abuse most frequently encountered in these cases, and examines the attitude of the courts to post-separation abuse by looking at both interim and final court orders. The study finds that key legal provisions governing these cases are not being followed, with little understanding shown for the nature of post-separation abuse.

## Introduction

Twenty years ago, an article by Humphreys and Thiara (2003) sounded a rare note of optimism in the otherwise consistently pessimistic literate on domestic abuse. They noted that, although court-ordered contact between a separated father and child was a likely context for the continuation of abuse perpetrated by that man against the child's mother, recent guidelines issued by the courts in England and Wales had the chance to “subvert abusive practices” given the need to ensure that the safety of both child and mother were considered before any contact could be ordered (Humphreys & Thiara, 2003, p. 209). This was not an unreasonable conclusion at the time—the guidelines in question did signal that courts were to change the way they approached post-separation parenting applications so that the risk of child contact being used as a means of engaging in further abuse was reduced (Lord Chancellor's Department, 2001). However, that optimism was misplaced.

This article presents the findings of a study of over 100 court files from England and Wales involving applications for post-separation parenting arrangements in which the parties identified as being at risk of harm. In particular, it examines those cases in which one of the parents (usually mothers) were subjected to domestic abuse after the separation took place, and the measures adopted by the courts in response. It finds that, despite changes in law and court practice designed to strengthen the obligation to ensure that contact between parents and children is safe before it can take place, courts routinely order contact with few, if any, workable safety measures.

The article first briefly outlines the legal framework involved as well as the methods employed during the study. It will then focus on the nature of post-separation abuse which is alleged to have occurred in these cases. It will then examine the frequency with which different safety measures were employed, first in the context of interim orders (temporary court orders made while the case is still progressing toward the making of a final order) and secondly where safety measures formed part of the final order made by a court. This article is the first attempt to systematically examine the nature of post-separation abuse allegations made in English and Welsh courts based on a case file review, and should, therefore, provide a useful evidence base for determining which kind of safety measures will be most suitable. The article will conclude that any optimism or faith in the ability of the current legal framework to adequately protect women and children from post-separation abuse was misplaced. Only a radical re-evaluation of the current position can lift the sense of despondency and futility which hangs over this area.

## Background to the Study

When parents separate in England and Wales, Section 8 of the Children Act 1989 provides that either parent can apply to a court for an order, known as a child arrangements order, which regulates where the child lives and who they spend time with. It therefore provides the legal basis for determining post-separation parenting regimes in circumstances in which the parents cannot come to an arrangement without court assistance. When making an order under Section 8, courts are obliged to regard the child’s welfare as the primary consideration, and to make their decisions utilizing the welfare checklist outlined in Section 1(3) of the Act. This lists a number of factors that the court must consider, including the child's needs, their wishes and feelings, parental capacity, and any harm the child has suffered, or is at risk of suffering. Further, an amendment passed in 2014 provides that courts are to presume that the involvement of a parent in a child's life is in their best interests unless the other parent can prove that involvement of any form would expose the child to the risk of harm (Section 1(2A) Children Act 1989). It should be noted, however, that this provision is being reviewed by the Ministry of Justice (Ministry of Justice, 2020), although there have been no findings from the review published to date. Research has highlighted serious problems faced by parents when they raise safety concerns (Hunter et al., 2020), with earlier reports showing the potential for fatal consequences when courts ignore these concerns (Women's Aid, 2016).

Research has regularly highlighted how applications under Section 8 consistently result in non-resident parents, usually fathers, being given substantial contact with their children, even in circumstances in which there is evidence of domestic abuse having been perpetrated against the mother, or even when such abuse may be ongoing (Coy et al., 2012; Harrison, 2008; Hester & Radford, 1996; Macdonald, 2016; Newnham & Harding, 2016). This is partly because of the attitude of the senior courts, which have posited that domestic abuse is not in itself a barrier to contact, and that the seriousness of the domestic abuse, the risks involved and the impact on the child must be weighed in every case against the positive factors, if any, of contact (*Re LVMH*, 2001). The legal position could therefore be summarized as follows: although there is no legal presumption in favor of contact, there is a legal presumption that both parents should continue to be involved in a child's life. Further, there is a cultural assumption that “involvement” means “contact,” leading to a belief that contact with a non-resident father should be granted; the presence of domestic abuse in a family's history may be sufficient to offset this assumption, but the threshold for doing so is high.

With the development of Practice Direction 12J in 2008, courts were given additional guidance on how to structure their decisions in cases where one parent alleged that harm to themselves or their child would result from contact. A Practice Direction is a type of court rule designed to regulate and standardize the ways in which certain types of court applications are conducted; as such they are binding on all courts in any situation in which they apply. Practice Direction 12J, which now forms part of the Family Procedure Rules, initially provided that the needs of the parent with whom the child ordinarily lived needed to be considered, as did any allegation of domestic abuse. However, this Practice Direction did not function as hoped and was revised in 2014 and 2017. These revisions were necessary because of the continued practice of granting unsupervised contact to abusive parents, often with disastrous consequences (Women's Aid, 2016); the revised Practice Directions were therefore designed to strengthen the protections afforded to resident parents. They permitted courts to hold separate hearings to deal with abuse allegations, and strengthened the obligation on courts to ensure that parents and children are not exposed to an unmanageable risk of harm, as well as widening the definition of domestic abuse to include coercive and controlling behavior.

Recognizing the social reality that risk cannot be entirely eliminated, the current version of the Practice Direction emphasizes that courts are obliged to engage in a process of risk minimization. It was envisaged that courts would assess the risk to both children and primary care-giving parents and allow contact with the other parent

only if it is satisfied that the physical and emotional safety of the child and the parent with whom the child is living can, as far as possible, be secured before, during and after contact, and that the parent with whom the child is living will not be subjected to further domestic abuse by the other parent. (Practice Direction 12J, [36])

Given the potentially protracted nature of legal proceedings, the Practice Direction also makes clear that, if a parent seeks contact on an interim basis while the proceedings are ongoing, the court should not make an interim order unless it is satisfied that it is in the interests of the child to do so and that the order would not expose the child or the other parent to an unmanageable risk of harm (Practice Direction 12J, [25]). To this end, courts are permitted to order that measures are put in place so as to guarantee the safety of contact. These can include specifying the location where contact is to take place, such as at a contact center, the supervision of contact or regulating the handover of the child from one parent to the other. It is also important to note that though a court may deem direct contact to be safe and beneficial for the child, it may still order that contact be supervised. However, in the event that a court deems that a parent seeking contact is a risk to the child or to the other parent, contact via a center or supervised by a relative is not appropriate (Practice Direction 12J, [38]).

The results of the present study support the findings of the Harm Panel Report which found that courts will grant as much contact, with as few restrictions, as possible (Hunter et al., 2020). This report is important because its examination of how courts dealt with domestic abuse and other harms occurred at a similar time as the present study, although using very different methods. The Harm Panel Report also found that there was a cultural assumption that contact between a child and a parent alleged to be domestically abusive would be ordered; where contact was initially limited or managed in some way, it would ordinarily progress to unsupervised contact. Abuse was minimized, allegations ignored, inadequate safety measures were adopted and inadequate assessments of risk were undertaken. It found that Practice Directions were not adhered to properly by courts. Crucially, it found that many professionals involved in parenting litigation do not understand the effects of post-separation abuse on mothers and children, and believed that it was irrelevant to litigation about contact. The present study adds significantly to these findings; at the time of the Harm Panel Report's compilation, no empirical work had been undertaken examining the operation of the revisions to court practices undertaken in 2017. The present study, therefore, brings the research up to date, confirming concerns outlined in the Report that legal changes are not working effectively.

Following the publication of the Harm Panel Report, some legal reform did occur. The Domestic Abuse Act 2021 was passed, but not all of its provisions have been commenced. Among the important changes it brought about are the revised definition of domestic abuse outlined in Section 1, and the recognition in Section 3 that children who witness domestic abuse are to be considered as victims in their own right. This is intended to serve as a reminder to family courts to take allegations of domestic abuse more seriously, given that abuse which is not directed at children must now be considered as if it were directed toward them (Bettinson, 2023). Further, it seeks to replace the complex series of orders currently available to victims of domestic abuse under the Family Law Act 1996 with a simplified set of remedies and provides victims of abuse with certain protective measures during family law proceedings. It should be clarified that none of these measures were in place at the time the study was conducted.

Despite ameliorating the situation for some victims engaging with the family courts, the Act failed to tackle some of the main problems experienced in that forum. For example, while it obliges that a report must be commissioned on the extent to which people using contact centers are protected from abuse, there is no legal obligation to take any action to implement recommendations of the report. Additionally, the presumption of parental involvement remains in place, meaning that both the legal presumption in favor of involvement and the cultural assumptions underpinning a pro-contact culture within family law are unaffected by the Act.

## The Study and Methodology

This study used case file research to gain an insight into the operation of Section 8 applications for child arrangement orders. Although relatively common in related areas such as social work where child protection files are examined, case file research is less common in the legal context because of the difficulties associated with accessing data held by courts and because of particular methodological challenges (Witte, 2020). The relative difficulty of gaining access is outweighed, however, by the benefits of accessing data which allows the study of a family's trajectory through the court system in a non-intrusive way. The process of examining case files whose primary function is not research can also tell us something about the various institutions involved, such as courts, child protection authorities, police and advocacy and support agencies; the personnel, institutional biases and preferences, and the pressures that these groups work under all become clearer. Thus, we cannot see the contents of a file as neutral and transparent, but as the result of a process subject to its own difficulties (Huskonnen, 2014).

Case files are thus inherently messy (Lucenko et al., 2015). The importance of this will be become apparent later in the article when the choices made by courts over how to respond to allegations of harm are discussed; depending on the choices made by courts and other actors, including the parties themselves, the contents of files may differ significantly even in ostensibly similar situations. Further, the content of the files will contain reports constructed by a mixture of professionals, designed to fulfill predetermined procedural functions and self-reporting narratives of both victims and perpetrators of abuse seeking to justify their choices. Therefore, the information contained in the files is further complicated by that report writer's own motivations (Fleming et al., 2015). Beyond this, there may be gaps in the data caused by missing information, different practices between different locations or professional bodies with result in similar incidents being recorded differently, and omissions caused by simple clerical errors. Each of these challenges was encountered during the course of this study, but ultimately their overall effect on the findings was limited.

In order to reduce the effect of information being missing from a particular report or the weight given to it being determined by a writer's motivations, the totality of information contained within a court file was accounted for in the coding of the data. For example, if an incident of physical abuse was absent from the initial court documentation but disclosed by later police reports, physical abuse was recorded as present in the case. Additionally, the study was largely exploratory in nature; the narratives or justifications of participants in legal proceedings did not significantly inform the analysis conducted, nor did they attempt to impute causation based on flawed or skewed perceptions of participants. This guarded against incomplete, partial or biased views informing the results.

This study examined case files relating to applications made under Section 8 of the Children Act 1989 issued between January 2018 and September 2019 in three Designated Family Court Centers. Permission to study the files was initially sought from HM Courts and Tribunals Service via the Data Access Panel in 2018, with permission finally being granted the following year. This required an initial Data Access Request followed by a Privileged Access Request which, given the nature of the information being sought, necessitated the approval of the President of the Family Division of the Royal Courts of Justice. Approval was then given for access to paper-based court files in three family courts.

The files were then examined on site at Designated Family Court Centers in November and December 2019. Anonymized data was extracted from each file, and each file was given a code number consisting of a letter, corresponding to the court center which dealt with it, and a number. A total of 684 cases were identified by HM Courts and Tribunal Service as being within the scope of the data access permission and as having a “risk of harm” flag, meaning that the Courts Service had determined that the 2017 version of the Practice Direction applied to each of the cases reviewed. Each application is assigned a file number when received by the courts, meaning that one family may have several linked applications if there are cross-applications from each parent relating to the same child, applications for variation of a previous order, or applications for enforcement of a previous order. When provided with the full list of 684 cases at the sampling stage, it was not possible to tell which applications related to the same family. It was only when on-site at the court center that it became evident that a particular application to which access had been granted had been physically consolidated with another, with one of these designated as the “lead application.” At the sampling stage, it was therefore possible that a group of four files would have been selected, but on arrival at the court center it could transpire that those four files related to a single family and had been consolidated into a single bundle for the purposes of court hearing dates.

A random sample of 50 cases at each court center was selected by the author for analysis, spread across the full date range; while this meant that some cases would not have been completed by the time of the site visit, it allowed for the possibility that some of these applications may have been connected to earlier proceedings or may have been dealt with in a relatively short space of time. Both possibilities were, in fact, borne out. Some incomplete cases also yield very important data on interim orders and other matters. A total of 14 cases were incomplete at the time of the study.

Due to time and resources constraints, including some ongoing hearings as well as the presence of linked or consolidated applications, it was not possible to examine the full 50 files at each location. Instead, a total of 102 lead applications were examined, spread as evenly as possible across the three centers. This represents 14.9% of all applications issued during the relevant period in these court centers. The selection process of these 102 cases was effectively random. Some case files which had been sought were not stand-alone applications, but were linked to other files which were also sought; as indicated above, it was not possible to determine whether this was the case at the initial sampling stage. This accounts for many of the files which were sought but were not designated as one of the 102 “lead” applications. A smaller number of files could not be examined simply because it was not available due to ongoing court business, a matter outside of the author's control.

Files were examined on site at three distinct Designated Family Court Centers. These centers do not only deal with cases from that city, but from their immediate environs as well. The first Designated Family Court Center (A) was located in a southern English city, characterized by a relatively younger and wealthier population than the national average. A lower percentage of children in that city live in low-income families and a higher percentage of adults have qualifications when compared to the national average. When looking at the wider catchment area, this relatively prosperous picture is maintained, as there is a higher life expectancy and lower levels of economic deprivation in this area than across the rest of England. The second Court Center (B) was located in a Welsh city with a slightly younger population than the Welsh average. The city and its surrounding area have experienced significant economic deprivation, and lag behind the rest of Wales in a variety of economic and educational markers, as well as health indicators.

The third Court Center (C) was located in a town south-east England. The town and its surrounding area measured comparatively poorly on educational disadvantage compared to national averages, but were mid-ranking on most other metrics relating to the economy and health. However, there was significant variation within the area covered by this court center, with some areas attaining standards significantly above the national average nestled very close to areas of significant deprivation. As a result, the study examined files from court centers that are broadly representative of the population across England and Wales. It was important to include a Welsh center in the study so that any potential variations between English and Welsh courts could be examined, although it was not feasible to include more than one due to resource constraints. Due to practical constraints, outlined below, it was not possible to attend court centers in other areas of England.

Due to data access constraints, the data could only be extracted from paper-based files; access to electronic files was not permitted by the Court Services. This immediately ruled out the possibility of accessing court centers which used electronic file management processes. No electronic equipment was permitted in the initial data capture on site, and the contents of files could not be dictated, copied or scanned. As a result, comprehensive handwritten notes were made about each file, which were later transferred to electronic format to assist the analysis. A mixture of quantitative and qualitative data was captured from each case file, which included the type and date of application, the risk of harm alleged by the parent applying for the order, any cross-applications or related applications, details of any expert reports, correspondence, interim orders and final orders. Where there were allegations of harm which were fleshed out via statements, reports, correspondence or other materials, this information was also captured. Some files included extensive information about earlier proceedings involving the same family, information about other generations or members of the same family, or occasionally documentation relevant to the case produced by other agencies. A chronological case history was created for each file examined, from which information relating to key variables was extracted and coded using SPSS, which was then used to generate descriptive statistics about the cases; the more qualitative data was also coded to identify different types of risks of harm and case outcome.

This study does, however, have some limitations. As already indicated, the restrictions on access prevented the examination of files from certain locations, such as the north of England, or a rural setting. The inclusion of additional centers meeting these criteria would have been desirable, but was not possible due to constraints. It is also the case that some information present in court files required a limited amount of interpretation by the researcher, thereby creating the possibility of bias in the interpretation of data. This was risk was increased by the lack of an inter-relater reliability study to ensure that any interpretation adequately reflected the contents of the files. The possibility of erroneous interpretation or bias in coding was guarded against in part by assessing the files in an exploratory way rather than seeking to prove a particular set of hypotheses. Furthermore, the existing literature on post-separation abuse was used to develop the variables being coded once the data had been collated. This helped to mitigate against the possibility of the researcher excluding particular forms of behavior from the coding process, thus reducing the potential for bias.

## Overview of Findings

### The Nature and Outcomes of the Cases

The findings of this study shed new light on the workings of courts dealing with Section 8 applications generally, and particularly those which involve domestic abuse. The data shows that the majority of applications were made by fathers. In total, 55.8% of applications were made by fathers, 41.1% by mothers, and the remainder were initiated by other family members, often grandparents who were also the children's special guardians, a position which confers parental responsibility on a person when a child cannot live with their parents. In the majority of cases, the children in the present sample were living with their mothers at the time of the application, and fathers were seeking spend-time-with orders, either to regularize the arrangements following an informal agreement, or were seeking the restoration of contact which had been stopped or reduced by either the mother or the children themselves.

Domestic abuse was alleged in 71.6% of all cases, with a remarkably consistent spread across all three court locations. The remaining cases concerned risks involving child abuse, or drug or alcohol addiction, while in a small number of cases, no further allegations of harm were made after the initial statement. The figure of 71.6% may not seem remarkable in itself, given that all of the cases examined in the study were flagged as involving an allegation that there was a risk of harm present in the case, but it is noteworthy. First, it shows how prevalent domestic abuse is in post-separation parenting cases, given that the sampled cases represent almost 15% of all Section 8 cases filed in the relevant period in the areas studied. Secondly, it is generally consistent with, and perhaps at the higher end of, previous estimates of the prevalence of domestic abuse in Section 8 cases. A range of studies undertaken between 2001 and 2017 generally placed the incidence of domestic abuse in contact cases at between 49% and 70% (Barnett, 2020). Direct comparison with those studies is not possible given the different methodologies involved including differences in the recording of information identifying risk of abuse and sampling. However, it is reasonable to infer that it broadly supports the findings of earlier work relating to the overall prevalence of domestic abuse concerns in post-separation parenting litigation. Further, it shows how domestic abuse is by far the most common form of harm identified in such cases.

The father was the sole alleged perpetrator in 78% of the cases where domestic abuse was alleged. Cross-allegations of abuse made by both parties featured in 20% of cases, with a single case involving a claim of abuse perpetrated exclusively by the mother. In 23.3% of cases involving domestic abuse, there had been police involvement with the family stemming from domestic abuse concerns, and in 14 cases (19%) the parties had been involved in earlier domestic abuse litigation resulting in an order under the Family Law Act 1996. Child protection authorities had also become involved, or were at present involved, in 51 cases in total, or 69.9% of those where domestic abuse had been alleged. Parental alienation allegations were often made by fathers in response to allegations of domestic abuse, with parental alienation claims featuring in a total of 17 cases, but only in three cases was it alleged by the father in the absence of a domestic abuse claim by a mother.

Despite the high level of animosity and clear risks to the safety of the families involved, the outcome of the cases examined in this sample was, almost inevitably, significant levels of unsupervised and overnight contact being granted by the courts. Out of a total of 102 cases, 93 lead to contact. Yet in addition to domestic abuse, the cases featured complicating factors including criminal convictions for serious offences, sectioning under the Mental Health Act 1983, and high levels of drug abuse. Despite these complex risk factors, the majority of cases not only led to contact but were concluded via consent orders, that is agreed between the parties with minimal, or no, judicial decisions imposed on the parties; effectively the court acts as a rubber stamp on privately negotiated arrangements. Of the 79 cases which were concluded with either an order on consent or a judicial order, 51 cases (64.6%) were resolved on consent, as opposed to 28 (35.4%) resolved via a judicially imposed court order. Therefore, in almost two-thirds of concluded cases, all of which involved a “risk of harm” flag and many of which contain allegations and perhaps clear indications of physical and sexual violence, the courts abdicated decision-making to the parties themselves. There was no evidence in the sample case files that consent orders were scrutinized by the court against the requirements of Practice Direction 12J beyond basic pro-forma statements.

In spite of the level of risk evident in the cases examined, contact was invariably the most likely outcome of a case, with only 9 cases out of 102 (8.8%) resulting in an order which would have permitted no contact; in one of these 9 cases, contact was not necessarily deemed inappropriate, but an order was considered futile as the child was about to turn 16 (Section 9(6) of the 1989 Act states that an order will cease to have effect once a child reaches 16). Such a finding shows that first instance courts are clearly operating within the framework of a pro-contact culture where contact would only be terminated in exceptional circumstances, where there are cogent reasons and there is no alternative. Yet the level of risk and harm which may be experienced by both the child and the parent with whom the child lives should serve to displace such a general principle, or at least allow for the creation of a new principle applicable to risk of harm cases. This is, in part what Practice Direction 12J was designed to do and appears to be failing to do in practice, proving the veracity of the view that the law is trapped in a cycle of failure (Hunter et al., 2018).

Within this group of 93 cases leading to contact, overnight contact was the end result in 50 cases, representing 53.7% of all contact outcomes, which is broadly in line with earlier research (Hunt & Macleod, 2008). However, given that all of the present cases involved an identified risk of harm, it would not be unreasonable to hypothesize that the figure would have been lower. This finding firmly supports the idea that there is a pro-contact culture, and that the factor most influential in driving decision making is not safety, but a desire to maximize contact.

### The Nature of Post-Separation Abuse

Legal literature in England and Wales has paid very little attention to the phenomenon of post-separation domestic abuse, with limited exceptions (Trinder et al., 2010). This is despite the fact that separation cannot be considered as a “vaccine” against abuse (Jaffe et al., 2003; Morrison, 2015), and that it has been integrated into the current understandings of domestic abuse which center on the abuse of power (Godsey & Robinson, 2013). The lack of attention given to post-separation abuse is especially troubling given that contact affords abusers court-ordered opportunities to continue their behavior (Khaw et al., 2018) and that contact handovers have long been identified as a significant context within which abuse occurs (Hester & Radford, 1996; Thiara, 2010).

Much the research that has been conducted on this topic is experiential in nature (Hay et al., 2023; Holt, 2017; Thiara & Humphreys, 2017), with little quantitative analysis. Research shows that common forms of abuse include verbal and psychological abuse (Fish et al., 2009; Kirkwood, 1993), harassment, stalking, threats of violence (including death threats), physical and sexual assaults (Kaye et al., 2003) and threats or attempts of suicide (Parker et al., 2008). Zeoli et al. (2013) outlines other behaviors regarded as examples of post-separation coercive control as including threatened or actual abduction of children (Harrison, 2008), undermining of mothers’ parental authority or capacity (Bancroft et al., 2012; Harrison, 2008), and using parenting time arrangements to track and control mothers’ schedules (Aris et al., 2002; Shalansky et al., 1999; Varcoe & Irwin, 2004).

The literature highlighted above served as the basis for the coding process and helped to isolate those cases which showed significant indications of abusive behavior. In some cases, it was evident that abusive behavior had ceased on separation, although as will be seen, this occurred in a minority of cases. In other cases, there was evidence of ongoing high interparental conflict. While the central theses of Johnson's (2008) work on situational couple violence are not accepted by this author, his labelling of certain behaviors as qualitatively different in nature and effect assisted this study's process of excluding from review those cases in the sample where isolated incidents of disagreement over a parenting or contact issue were mentioned during litigation. This choice was informed by the characterization of post-separation abuse as a continuation and escalation of pre-existing power and control dynamics. While the decision in this study to characterize certain cases as conflict-based rather than abusive does risk under-reporting the real incidence of post-separation abuse, it was necessary so that the focus could be placed on the courts’ responses to those cases where ongoing abusive behavior was most evident.

In total, 52 cases showed indications of post-separation abuse. Given that domestic abuse featured in 73 cases in total, post-separation abuse was a feature in 71% of cases where domestic abuse was alleged to have occurred and in just over half of all cases examined during the study. This is an extraordinarily high figure compared to the relative lack of attention the issue receives in both research literature and court decision making. Of these 52 cases, 42 (80.7%) were cases where the mother was the victimized parent. Of the remaining 10 cases, 6 involved cross allegations by both parents. One case involved an alleged abduction threat by a mother who had sole care of the child and who was not in any durable relationship with the father prior to, or after the birth of the child, two cases where abusive behavior was perpetrated by women with additional alcohol or drug addiction issues, and a single case concerned a mother was abusive with no additional or mitigating factors. In cases where cross allegations were made, the abusive behavior by each parent was often qualitatively different. In these cases, the behavior of women tended to be directed toward the removal of the children to another location (and so classified as threatened abduction in a legal sense) so as to ensure her or the children's safety in response to direct assaults, threats, harassment or coercive and controlling behavior, whereas the father's behavior frequently involved significant levels of violence and aggression.

In each of the 52 cases where post-separation abuse was observed, the abusive behavior was then coded into the following categories—stalking/harassment, physical/sexual abuse, emotional/psychological abuse, financial abuse, threats to the child/other parent, threats of self-harm, abduction or non-return of the child or threatened abduction, criminal damage to property, coercive and controlling behavior, and other. These categories were chosen because of their prevalence in the existing literature as indicators of post-separation abuse.

Some difficulties were encountered, however, given the potential overlap between categories. Threats of harm could easily be considered emotionally abusive, but an attempt was made to code any behavior which could constitute a threat as threatening behavior, while emotional or psychological abuse tended to show itself as insulting or belittling behavior designed to undermine a person's sense of self-worth. Coercive control could have been a catch-all term for virtually all of the behavior observed in the study; in order to lend greater precision to the coding, behavior coded as coercive control included exerting high levels of control over contact arrangements, late cancellations of contact, attempts to interfere in the child's education, malicious referrals to child protection authorities and attempts to undermine the relationship between the child and the other parent. Abduction, non-return of children and threats to do this were the most problematic category given it includes behavior ranging from the actual removal of children to another place without the consent of the resident parent to the lack of compliance with a court order to return a child at a specific time, to allegations that removal had been threatened. Despite the wide range of behavior, it was coded together with “abduction” because the legal responses to these different actions would be broadly similar. The results of this are presented below in Table 1.

**Table 1.** Types of Post-Separation Abuse.

	Center A (*n* = 21)	Center B (*n* = 14)	Center C (*n* = 17)	Total (*n* = 52)
Harassment	6	8	10	24
Physical	6	6	8	20
Emotional	3	4	3	10
Financial	3	3	3	9
Threat to self	1	1	1	3
Threat to others	6	5	6	17
Abduction	8	5	6	19
Criminal damage	3	3	3	9
Coercive control	11	6	10	27
Other	3	0	1	4

As can be seen from the data above, coercive control, harassment or stalking were the most common forms post-separation abusive behavior closely followed by physical abuse, abduction and threats of harm, confirming the assertion by Katz et al. (2020) that many abusers do not resort to overt violence in the aftermath of separation. Most forms of abuse in the present study were quite evenly distributed across all three court centers, showing how common these were regardless of geographic, socio-economic, or demographic variables. Exceptions, however, do occur. Harassment and stalking appeared to be less common in Court Center A, which was the most affluent area examined. It also had proportionately fewer incidents of physical or sexual abuse. This area also, however, had a disproportionate incidence of abduction or threatened abduction and non-return of children than other areas, perhaps due to greater opportunities for physical mobility. Behavior falling within the “other” category was most frequently observed here as well. The behavior included alleged child endangerment, child sexual abuse committed by the non-resident parent, and possession of pornographic material depicting children by a non-resident parent.

Whether these differences are because of a real difference in the forms of abuse found in this area, a difference caused by the way in which the behavior was reported by the parties involved or a random difference due to sampling cannot be known for certain. A difference in reporting behaviors of this scale across regions is possible, but not probable, given that these accounts are not based exclusively on self-reporting by victims. The study also includes reports from various statutory agencies who use standardized forms and reporting approaches, leaving random sampling difference as a more likely explanation. This is supported by the findings of a Fisher exact test which was conducted on the data. Each of the forms of post-separation abuse was cross-tabulated against the court center in which they were found. The *P*-values of these tests ranged from .221 for harassment to 1 for threats to self. Clearly, with an *α* value of .05, the hypothesis that geographical location had no effect on the frequency of a particular form of abuse being committed cannot be discounted. In order to understand whether the spread of all behaviors over the entire sample was likely due to chance, a Cochran *Q* test was performed on the whole sample. This returned a *P*-value of <.01. The *α* value for this test was also set at .05, meaning that this result is statistically significant, and the general tendency of certain behaviors to occur in certain regions is not due exclusively to chance. The true explanation will, however, have to wait until more extensive work is carried out on this topic; the sample size, though significant, is probably not sufficient to provide an adequate explanation.

### Safety Measures in Interim Court Orders

In order to be effective, safety measures must address the particular risks which are present in an individual case. While this may seem obvious, there is little evidence from existing literature, or indeed from the present study, that courts are taking anything akin to a tailored approach to safety measures. Indeed, they seem particularly ill equipped to handle non-physical abuse such as stalking and harassment or coercive control. Given the prevalence of these forms of abuse, safety measures must be addressed to tackle alleged abusers’ “high sense of entitlement and self-centeredness,” which can result in harm not only to the resident parent but influence their fathering practices (Bancroft et al., 2012; Humphreys et al., 2019).

Given the potentially protracted nature of legal proceedings, Practice Direction 12J is clear in stating that where there are allegations of domestic abuse which have not yet been determined to be true or false, a court should not make an interim order permitting contact unless it can ensure that the child or parent will not be exposed to an unmanageable risk of harm. If it decides that the risk is manageable, the court should consider how to do so by the imposition of appropriate safety measures, such as the supervision or supporting of contact, or whether indirect contact is more appropriate. In some cases, multiple interim safety measures will be ordered. For example, a court may order that contact be supervised and at the same time grant an order, such as prohibited steps order, which will prevent the parent against whom it is made from taking a particular action, such as removing the child from school. However, interim safety measures may also be structured so as to facilitate the transition from supervised to supported contact, or to unsupervised contact (Table 2).

**Table 2.** Outlines the Range of Interim Safety Measures Imposed by the Courts (*n* = 52).

Supervised contact	14 (26.9%)
Supported contact	3 (5.8%)
Indirect contact	1 (1.9%)
No contact	4 (7.7%)
Managed handover	5 (9.6%)
School handover	5 (9.6%)
Prohibited steps order	8 (15.4%)
Undertaking	6 (11.5%)
Non-molestation order	2 (3.8%)
Other measures	11 (21.2%)
No safety measures	16 (30.8%)

As can be seen, the most likely outcome was for some form of managed or restricted contact, which is taken to mean supervised, supported, or indirect contact, closely followed by no safety measures at all. While at first glance, this seems to indicate that safety is being taken seriously, it does not show how many of these cases with restricted contact were envisaged as having unsupervised or unrestricted contact as a natural progression. Neither does there appear to be any significant engagement with the different forms of harm which are particularly associated with post-separation abuse, nor any evidence that the form of abuse influenced the interim order made.

It has previously been found that supervised and supported contact was usually used by the court as a stepping stone to unsupervised contact, rather than as an endpoint in itself (Newnham & Harding, 2016), meaning that supervision is seen as a temporary arrangement. In the present study, this happened in two cases within the lifetime of the interim order. This means that while supervised contact was deemed an appropriate safety measure in the immediate term, the court envisaged that contact would “progress” to being supported or unsupervised without any further findings being made or any positive actions being taken by the parent deemed to be a risk. This clearly calls into question the court's motivations in imposing a restriction on contact in the first place; more than likely, an order for supervised contact was made so as to be seen to assuage the resident parent's concerns.

It should also be noted that 7 of the 14 instances of supervised contact were ordered in Court Center A, despite the fact it accounted for only 40% of cases of post-separation abuse. This cannot be accounted for solely by the type of risk involved, as this center, as indicated above, had a lower rate of risk of physical abuse and stalking/harassment than the other centers. Similarly, it cannot be due to the number of facilities for supervised contact available in the immediate environs of the court center, as similar services are available at a similar level in each center examined. The only explanation appears to be judicial preference, indicating the presence of a postcode lottery for women and children at risk of domestic abuse; those living in a particular district are more likely to be afforded supervised contact as a safety measure simply because of where the case is heard.

In situations where supervision was deemed appropriate, contact supervisors were most commonly family members of the parties, and of the alleged perpetrator of abuse in particular. In one particular case, the father was accused of sexual abuse of a child who was not subject to the present proceedings. When the mother objected to contact because of these allegations, the court ordered that the paternal grandmother act as supervisor, despite her express statement that she did not believe the sexual abuse claim. The attitude of the courts to safety measures could, therefore, legitimately be described as desultory and contrary to the express statement in paragraph 38 of the Practice Direction that contact supervised by a relative is not appropriate.

The cases in which no safety measures were ordered also require further examination, alongside those cases which utilized undertakings as safety measures. No safety measures were adopted in 16 cases. The most common form of abuse in these cases was stalking and harassment, which occurred 9 times, followed by physical abuse and abduction/threatened abduction (4 cases each), financial abuse and threats to the child or resident parent (three cases each), including one where the child herself believed that her father would kill her. Much the same pattern was repeated in cases involving undertakings. Undertakings are, in effect, promises given to a court to do or abstain from doing a particular action. Despite not being court orders, they can be legally enforceable, although in the sample of cases examined, there were no instances of an application for enforcement of a broken undertaking. As a result, they took on character of promises or wishes rather than binding rules or directions, despite their legal status as such. Of the six cases in which undertakings featured, they were the sole safety measure adopted in three cases. In looking at all cases with undertakings, all save one featured some combination of harassment, coercive and controlling behavior, physical or sexual assault, financial abuse, and threatened abduction.

These findings are of vital importance. Women's Aid (2016) examined the cases of 12 men who had killed their children after parental separation; 9 of these men had committed domestic abuse after separating from the child's mother. They had engaged in exactly the types of behavior which in the present sample led to the imposition of no interim safety orders at all, or voluntary promises of better behavior which were routinely unenforced. It is evident that the courts under examination have not learned the lessons of earlier tragic cases.

### Safety Measures in Final Court Orders

Before examining the content of final court orders, it must first be reiterated that, due to the ongoing nature of some of the cases under review, not all cases in the sample had concluded at the time of the study. On occasion, the parent against whom safety measures were ordered withdrew from the case, as it was obvious that unsupervised contact would not be grated to them; this happened in four cases with the parties reconciling in one further case. Of the 52 cases with post-separation abuse, eight were not completed, which when combined with the withdrawn and reconciliation cases, reduces the sample to 39. Of these, a safety measure of some form was adopted in 17 cases (43.6%). Of these 17 cases, four featured a safety measure as part of the final order where no interim safety measures had been ordered. This may have been because there had not been an interim hearing, or the court was waiting for further reports or information before deciding on whether safety measures were required. Conversely, 81% of cases with safety measures in the final order also had interim safety measures, thereby demonstrating that the chance of having an ongoing safety measure put in place by the courts is greatly enhanced by having the courts agree that one is required on an interim basis.

The most common safety measures to feature in a final order were the attachment of conditions relating to the handover of the child or children for contact. This type of measure was ordered in 11 of the 17 cases (64.7%). These conditions varied considerably in detail, ranging from supported handover, which was likely to involve a neutral third party such as a contact center provider, to handovers having to occur in public places covered by CCTV. Occasionally, third parties, such as new partners or grandparents would be barred from handovers. However, the most common provision, found in 5 of the 11 cases with handover provisions, was that indirect handover was to occur via the children's school or nursery.

The logic of such orders appears to be that by having handover occur in this way, the parents did not need to meet, thereby reducing the risk to the vulnerable parent. However, it is hard to see how managed handovers would meaningfully provide greater safety. In the cases with safety provisions, the post-separation abuse included staking and harassment (which often occurred electronically), malicious referrals to child protection agencies, financial abuse through the non-payment of child support, criminal damage to the mother's home or other property, and threats to harm, abduct or kill delivered electronically or via the children. Managed handovers can in no way materially address these forms of abusive behavior. Instead, they appear to represent a willingness to be seen to do something which ostensibly protects the safety of the families involved, while at the same time not materially affecting the contact enjoyed by the non-resident father.

Managed handover could also occur via a contact center. Yet serious safety concerns arise even when such orders are made. Recent research on safeguarding in contact centers has highlighted that only 11% of staff had received specialist domestic abuse training in the past 12 months, despite contact centers being increasingly utilized as a key protective measure in high-risk domestic abuse cases (Ministry of Justice, 2023). Additionally, contact centers appear to have a high threshold for determining when incidents occurring at contact sessions warrant intervention, leading center staff to focus on events involving physical altercations rather than emotional harm. The behaviors outlined above which occur outside of the contact center would also fall outside the scope of that center's remit. Therefore, only a relatively small range of actions are within the power of the center to stop, and such events are often treated as individual incidents rather than as part of a pattern of coercive control (Ministry of Justice, 2023), thereby limiting their ability to satisfactorily protect families with a history of domestic abuse from being revictimized through contact. Nonetheless, contact centers at least provide some measure of independence and a safety net for parents and children during the contact process that handover via relatives or schools cannot.

Curiously, none of the cases which provided for handover to be managed by relatives were heard in Court Center A. Here, handover was managed exclusively via centers, by stipulating that no one other than the parents were to be present, or by expressly excluding a specific person. No such orders were made in Court Center B, and only one in Court Center C. This is a significant regional variation, the cause of which is unclear. Given the significant differences in approach, it is unlikely to be due solely to sampling. More likely, it reflects a greater judicial willingness in Court Center A to use contact centers. However, this calls for further explanation. The most likely reason, taking the demographics of the areas surveyed into consideration, is that judges in Centers B and C realize that the families involved may have been less able to afford such services than families in Center A, or that there may be greater financial support available in Center A for families utilizing these services.

This is borne out by the recent research on contact centers, which highlighted that the cost to families ranged from £40 to £100 per hour for supervised contact, a cost described as inaccessible for many families (Ministry of Justice, 2023). Once again, this raises the specter of a postcode lottery affecting vulnerable families. If a particular case's facts do indicate that managed handover is an appropriate safety measure, courts in areas which do not routinely favor the specialist management of handover will place an undue burden on already overstretched schools and childcare providers who are not necessarily equipped to manage the relevant risks. Alternatively, they may rely on potentially biased relatives of alleged abusers, in direct contravention of the Practice Direction. Further, it is deeply concerning that the level of protection afforded to women and children from domestic abusers differs according to their own means or the means of either local government or the voluntary sector to provide financial assistance for specialist handover management. This merely exposes the already financially marginalized to avoidable risks.

The next most common form of safety measure found in final orders related to the management of contact itself. Managed contact here refers to a variety of different mechanisms—support, supervision, or indirect contact—designed to deal with safety or welfare concerns. Orders which included provision for managed contact featured in 7 cases—4 from Center A and 3 from Center C, with none at all in Center B. In Center A, three of the cases resulting in managed contact involved allegations of physical assault while the other involved threats to kill the children. The clear inference to be drawn from this is that courts will only restrict contact if there is a clear indication of physical, as opposed to emotional harm, to the mother or children. This, however, is not entirely supported by the data available from Center C. Of the three cases involving managed contact, two involved an allegation of physical assault or threats. All, however, involved substantial levels of stalking and harassment. Other salient features which likely influenced the decision to order managed contact include threats to remove the children from the resident parent, enrolling the children in new schools without the resident parent's consent, and breaches of earlier court orders. All cases which resulted in managed contact did, therefore, involve serious safety concerns but, as will be discussed below, not all serious safety concerns resulted in managed contact.

Courts can, and do, use final orders to provide pathways out of managed contact so that it can gradually become unmanaged, overnight contact, as indicated in the previous section. Of the seven cases with managed contact, only three were envisaged as remaining managed for the foreseeable future, meaning that in four of the seven cases, the court ordered that the contact would be unsupervised within a matter of months, or more frequently, weeks. Of these four cases which progressed to unmanaged contact, only one was the result of a fully contested hearing, with two being consent orders based on agreements between the parties, and one where the father did not even attend the hearings.

In the three cases where longer term management was envisaged, these can be characterized as factually unusual; in one case, the child expressed a clear preference for indirect contact only, having witnessed the physical assault of his mother by the father and having been the subject of the father's emotional abuse himself. In another the father was permitted supervised contact on an ongoing basis, even though he had broken into the former family home, where the police and fire services had to install anti-arson measures; he had also threatened to kill himself in front of the mother, abducted the youngest child from her nursery and attempted to abduct the other children from school. He was also found to be in possession of over 1,200 extreme illegal pornographic images, over half of which were stated by the police as being Level 5 images, meaning that they depicted a sexual image of a child in a situation which involved “sadism or penetration of, or by, an animal” (Sentencing Council, 2002). That contact was allowed to continue in these cases, even though it was managed, and shows an extraordinarily limited understanding of the nature of the risks posed by some fathers and how deeply ingrained the pro-contact culture is among judges.

Finally, there were eight cases where a safety measure was part of an interim order, but not part of the final order. In one of these cases, the parties reconciled following the interim hearing. Of the remaining seven, one saw the interim restrictions lifted following the closure of child protection investigations, and five saw the parties agree on unmanaged contact by way of a consent order. The one remaining file was unclear as to whether the order was on consent or not. This high number of consent orders is crucial for understanding the lack of attention paid to safety concerns in many cases. Four of the five cases involved allegations of physical assault on the mother by the father, with two of these women claiming that they were raped by their ex-partners. Nonetheless, despite courts ordering some restriction on contact as an interim measure, the courts accepted as legitimate the agreement made between alleged abuser and victim. In these cases, the courts simply rubber-stamped the agreement, paying no attention to the ongoing trauma, or the power imbalances inherent in the dynamic between the parties. Previous research has highlighted how courts are often under pressure to speedily resolve cases, which can lead to women acceding to arrangements that are not necessarily in their or their children's interests (Coy et al., 2012). This is compounded in the cases involving sexual abuse; for courts to simply accept agreements made between an alleged rapist and his victim, without any attempt to establish the merit of the complaint or to properly inquire into the impact of contact on the safety and wellbeing of the mother and children, is tantamount to a dereliction of duty.

## Conclusion

This article began by highlighting that the optimism which was sometimes felt at the beginning of this century for the future handling of contact cases involving domestic abuse. The development of new guidelines and pronouncements from senior judges that domestic abuse was an important factor to consider when assessing a child's best interests gave a legitimate cause for hope that the safety of parents and children would no longer be side-lined as part of a contact at all costs culture. Sadly, that optimism has been shown to be misplaced. Literature since that time highlighting women's experiences of the court system shows a minimization of domestic abuse even amid significant procedural changes designed to do the opposite. The study on which this article is based adds to that literature by using case file research to show how prevalent post-separation abuse really is in child contact cases. It has further shown how, rather than engage in the mandatory process of risk minimization, it appears that courts are really conducting an exercise in the minimization of abuse narratives. It has done so by highlighting evidence of the inconsistent and inappropriate use of safety measures in interim court orders, with safety measures often being used as a stepping stone to a normalized outcome of unsupervised contact with a parent who poses significant risk to the other parent and to the child. Further, it highlighted how, by adopting measures ranging from the use of undertakings by alleged abusers and supervision of contact by those abusers’ relatives, to ordering schools to facilitate the handover of children, courts are displacing the responsibility for ensuring safe contact onto inappropriate or ill-equipped third parties.

These findings show a clear dissonance between what the law demands and what is being accomplished in courts. It is now clear that decades of evidence showing the need for a culture change have not resulted in such a change taking place among local level courts tasked with the vast majority of family law litigation. The level of abuse which parents, usually mothers, are expected to tolerate during, and after, separation so that their ex-partner retains direct, unsupervised contact with his children presents a legitimacy problem for the law because it is simply failing to do what it says it will do—ensure that contact is safe for all concerned. The outcome of this cannot be further calls for cultural change—that has been tried for over 20 years, and failed, bringing with it a sense of despondency and futility (Birchall, 2022). The presumption of parental involvement is under review; a radical reappraisal of who contact is for, why is it granted, and how it works is required as an essential part of that review if any progress toward safe contact is to be achieved.
